# Path analysis of skin cancer preventive behavior among the rural women based on protection motivation theory

**DOI:** 10.1186/s12905-020-00978-8

**Published:** 2020-06-11

**Authors:** Nasrin Roozbahani, Abdol-Hossain Kaviani, Mahboobeh Khorsandi

**Affiliations:** Department of Health Education & Promotion, School of Health, Arak University of Medical Science, Arak, Iran

**Keywords:** Protection motivation theory, Skin cancer, Rural women, Iran

## Abstract

**Background:**

Determining the effective factors on the adoption of preventive behaviors capable of reducing the risk of skin cancer is an important step in designing interventions to promote these behaviors. Based on the protection motivation theory, the present study is aimed to conduct a path analysis of skin cancer preventive behaviors in rural women to explore these factors.

**Methods:**

In this cross-sectional study, 243 rural women were randomly selected from the west of Iran to receive a valid and reliable questionnaire assessing constructs from the protection motivation theory, as well as demographic information. Fully completed questionnaires were returned by 230 women and the data were analyzed by SPSS 22 and LISREL8.8.

**Results:**

Concerning skin cancer preventive behaviors, 27.8% of women wore sun-blocking clothing when working under the sun, 21.7% used sunscreen cream, 5.7% wore a cap, and 4.8% used gloves and sunglasses. Protection motivation theory and per capita income explained 51% of motivation variance and 25% of the variance of skin cancer preventive behaviors. The response efficacy construct was the strongest predictor of the motivation of protection (ß = − 0.44, *p* < 0/001). Per-capita income (ß = − 0.34, *p* < 0/001) and motivation (ß = − 0.33, *p* < 0/001) were the strongest predictors of these behaviors.

**Conclusions:**

This study showed that protection motivation theory is efficient in predicting skin cancer preventive behaviors and the interventions can be designed and implemented by this theory. Proper planning is also necessary for promoting these behaviors among people with low per-capita income.

## Background

Skin cancer is the first and second common cancer among Iranian men and women, respectively [1]. In skin cancer, the epidermal layer of the skin grows abnormally. This type of cancer is classified into melanoma and no melanoma. Skin cancer has an increasing trend and 2–3 million people are globally affected by this disease [2, 3]. The main environmental risk factor for skin cancer is the ultraviolet (UV) radiation emitted from the sun and other sources. The evidence has indicated that self-examination of skin lesions and behavioral counseling could have a unique role in early diagnosis and cancer prevention [4, 5].

Although skin cancer is one of the most prevalent cancers, it is one of the most preventable ones as well [6]. In other words, effective factors such as race, heredity, skin color, and genetic background may not be changeable, but public awareness and changeable factors can be improved through public health educations [7].

Individuals who work hours of daytime under the sun are more susceptible to skin cancer. As one of the high-risk groups, rural women have to work for many hours under the sun. Without appropriate protection against UV radiation, they are highly susceptible to skin cancer. This group should take protective measures such as limiting outdoor work hours, avoid sunlight exposure from 10 am to 4 pm, and wear protecting equipment such as a wide-brimmed hat, long sleeve dress, and sunscreen with a protective factor (SPF) ≥ 15 [8].

High skin cancer prevalence along with its corresponding mortality, and disability, as well as emotional and physical suffering, have necessitated the implementation of prevention measures. In this path, most advancements can be achieved when, in addition to the recognition of the present situation, the effective factors on the behavior are also considered. One of these factors is the individuals’ motivation to implement risk-reduction behaviors. In this regard, protection motivation theory (PMT), as one of the effective theories in health education, provides a unique framework to predict health behaviors. This theory assumes that the adoption of healthy behavior (a protective behavior), recommended against a health risk factor, is a direct action of the individual’s motivation to protect himself [9].

This theory provides a framework for understanding fear and the ways people try to protect themselves against health threats. PMT is originated from the results of threat appraisal and the coping appraisal. Threat appraisal includes perceived vulnerability (a person’s belief that he/she is vulnerable to a health threat), perceived severity (a person’s belief that health threats are severe and serious) and perceived rewards (rewards that a person receives from doing unhealthy behavior or not doing healthy behavior). Coping appraisal includes perceived self-efficacy (a person’s belief of performing healthy behavior successfully), response self-efficacy (a person estimates that healthy behavior works), perceived costs (a person estimate on the costs of protective behaviors). Fear resulted from these two appraisals creates the motivation to perform health protection behaviors [9, 10].

Studies on PMT have indicated that its constructs have high importance in predicting cancer-preventing behaviors [11–13]. Due to challenges in motivating women to participate in cervical cancer screening, Bai et al. in china studied the role of PMT in predicting their tendency toward performing cervical cancer screening [14]. They concluded that focus on cancer knowledge, awareness, and previous experience regarding screening and demographic factors are associated with the screening tendency through promoting cancer risk perception and reducing response cost [14]. In another study, Rahaei et al. assessed the predictors of cancer early detection behaviors using PMT. They indicated that PMT constructs are useful in predicting protection motivation, and passive and active behavior in the cancer early detection initiatives [13].

According to the above discussions, and the importance of rural women’s health and lack or inadequate local and international evidence in this regard, this study was designed to perform path analysis of skin cancer preventive behaviors among rural women in the west of Iran based on the protection motivation theory.

## Methods

### Participants and procedures

This cross-sectional study was carried out in 2017 among rural women of Nahavand, a city in the western part of Iran in Hamedan province with a population of 72,000. It should be noted that villages in Iran are covered by cities based on geographical divisions. So, if a researcher wants to perform a study on villages, he/she should at the first select the considered cities.

This city was selected using a random digits method from the list of all cities in the west of Iran. There are 43 cities in the west of Iran located in Kermanshah province (14 cities), Kurdistan province (10 cities), and Hamedan province (10 cities) [15]. As the people living in cities located in the west of Iran have a similar cultural, economic, and social status and a somewhat common language and they live under the same climate and sunlight from one hand, and regarding the limited available research resources, on the other hand, it was decided to consider only one of the mentioned cities.

Another important issue is that rural women are usually exposed to the sun while performing household affairs. In other words, many of the affairs near and outside houses are the duties of rural women.

The rural population refers to people living in rural areas as defined by national statistical offices. A rural area is a geographic region located outside towns and cities. Villages are often located in rural areas. In other words, all populations, housing, and territory not included in an urban area compose villages [16]. Through the cluster sampling method, 4 villages were randomly selected from Nahavand city. Then, using the documents of health centers located in the villages, the women were selected through a random sampling method. All demographic information of the Iranian rural population was recorded in health centers. Rural health centers provide this information through annual census by their employees. This operation is supervised by district health authorities. Such statistics can help planning and developing primary health care in rural areas [17].

The lowest sample volume by attention to the previous studies [11, 14], considering the maximum standard deviation of 5.4, acceptable error of 0.7, and confidence interval of 95%, was estimated 230 people using n = z2s2/d2 formula. Moreover, according to Kock et al. study on minimum sample size estimation in the least squares-based structural equation modeling (PLS-SEM), the minimum sufficient sample size is 160. On the other hand, 1628 women met the inclusion criteria in the 4 selected villages. The eligible women were selected through simple randomized sampling proportional to the village’s population. Therefore, 243 women entered the study and received the questionnaire. Lastly, 230 women returned fully completed questionnaires [18].

The written informed consent form was also collected from the participants. This form explained the purpose of study, expected duration of the subject’s participation, a description of the procedures, risks or discomforts and benefits, confidentiality, and a statement regarding voluntary participation and freedom to leave the study at any time [19]. If one of the selected subjects was not willing to participate in the study, another person was invited instead.

Inclusion criteria were rural women with minimum literacy or above, older than 18 years old, who had not been diagnosed with skin cancer. The exclusion criteria were as follows: partial presence at the training sessions and the tendency to leave the study. The training sessions regarding the importance of the study, how to answer the questions, freedom to leave out the study were separately held for each participant which lasted for about 20 min.

### Measures

The study instrument included a standard questionnaire for skin cancer based on PMT which has 2 sections of socio-demographic variables and PMT theoretical constructs [20]. The participants were interviewed by one of the research team members at their homes.

The socio-demographic variables included age, gender, marital status (single/ married/ widow), education level (illiterate/ elementary/ secondary/ high school/ diploma/ college degree), job (household/ worker/ employee/ self-employment), number of hours working under sunlight, history of sunburn, number of family members and the family monthly income level. The existence of a cancer patient in the participants or their relatives was also asked.

The second part of the questionnaire included questions measuring PMT theoretical constructs including perceived vulnerability (e.g., If I have been exposed to sunlight for a long time, my skin will be damaged) (4 items), perceived severity (e.g., Skin cancer is not too concerning) (3 items), perceived rewards (e.g., It’s a pleasure to be under the sunlight) (3 items), perceived fear (e.g., I feel bad about skin cancer) (3 items), perceived response (e.g., If I use cap and sunglasses, I can reduce the risk of skin cancer) (3 items), perceived costs (e.g., It’s time-consuming to wear a cap and sunglasses) (6 items), perceived self-efficacy (e.g., I can prevent skin cancer) (5 items) and protection motivation (e.g., I decided to be less exposed to sunlight) (5 items) and also skin cancer preventing behaviors (8 items). The responses to the theoretical constructs were scored using a 5-point Likert scale ranging from 1 (strongly disagree) to 5 (strongly agree). The responses in the behavior assessment questions were scored ranging from 0 (never) to 4 (always). Some questions were scored reversely.

### Validity and reliability

To confirm the fact validity of the research tool, 10 experts reviewed the level of difficulty, the extent of inappropriateness, phrase ambiguity, and failure in the meaning of words, and recommended their corrections.

To assess content validity, a panel of experts consisting of 10 university professors in the area of health education were asked to assess the questions quantitatively and qualitatively. In the qualitative method, the experts were asked to assess the questionnaire grammatically compliance and evaluate the right wording, proper items organization, and scoring. Finally, their feedbacks (mainly related to the wording and phrasing of the items) were used to revise the tool.

In the quantitative method, content validity ratio (CVR) and content validity index (CVI) were confirmed. To this end, 15 experts were requested to state their views for each item on a three-degree scale “it is necessary”, “it is useful but not necessary” and “it is not necessary”. Given the number of experts (15 people), based on the Lawshe table, the CVR amount should be 0.49 to confirm its content validity. As CVR for all questions was higher than 0.49, content validity was confirmed.

To assess CVI, the experts reviewed the relevance, simplicity, and clarity of each item. The results were applied in the questionnaire. The questionnaire reliability was assessed through Cronbach Alpha on 40 rural women with similar demographic characteristics with the study population. The questionnaire Cronbach Alpha was higher than 70%.

### Path analysis

Path analysis was used to assesses PMT and predict the preventive behavior of skin cancer. The used indices were *χ*^2^ whose insignificant amount indicates theoretical fitness with the data, the ratio of *χ*^2^ to the degree of freedom in which the amount lower than 3 is preferred, and comparative fit index (CFI), the goodness of fit index (GFI), adjusted goodness of fit index (AGFI), normed fit index (NFI) whose amounts higher than 0.9 were favorable for all these items. Regarding root mean square error of approximation (RMSEA) and root mean square of residuals (RMSR), the amounts lower than 0.05 were very good and 0.08 were acceptable [21].

### Statistics

The collected data were analyzed using SPSS 22 and LISREL8.8 through the intraclass correlation coefficient, maximum likelihood method, and correlation matrix. The linear structural relations model (LISREL) was also employed to determine whether the data fit the model or not.

## Results

The mean age of the participants was 30.55 ± 7.50, mostly educated to elementary level (42.6%), and most of them were housekeeping (87%) and married (87.8%). The job and education level of most of the participants’ husbands were manual workers (28.7%) and elementary school (29.6%), respectively. Most households’ monthly income was lower than 125 USD. The mean working duration under the sun was 2.72 ± 1.46 h, and most of the participants (67%) had a history of sunburn (Table 1).

**Table 1:** Demographic information of the rural women participated in path analysis of skin cancer preventive behaviors using PMT

Variable		Number (percent)
Marital status	Single	24 (10.4%)
	Married	202 (87.8%)
	widow	4 (1.7%)
Education level	Illiterate	16 (7%)
	Elementary	98 (42.6%)
	Secondary	42 (18.3%)
	High school	44 (19.1%)
	Diploma	23 (10%)
	College	7 (3%)
Job status	Housekeeper	200 (87%)
	Farmer	4 (1.7%)
	Rancher	3 (1.3%)
	Other	23 (10%)
Job of their husbands	Without husband	28 (12.2%)
	Farmer	50 (21.7%)
	Rancher	4 (1.7%)
	Employee	17 (7.4%)
	Worker	66 (28.7%)
	Other	65 (28.3%)
History of sunburnt	Yes	154 (67%)
	No	76 (33%)

*PMT* Protection Motivation theory

Regarding skin cancer preventing behaviors, 27.8% of the participants always wore sun-blocking clothes, 21.7% used sunscreen, 5.7% wore caps and 4.8% of them used gloves and sunglasses (Table 2).

**Table 2:** Skin cancer preventing behaviors in the rural women participated in path analysis of skin cancer preventive behaviors using PMT

	Number (percent)				
	Never	Sometimes	Half of times	Most of times	always
Wearing caps	36 (15.7%)	71 (30.9%)	62 (27%)	48 (20.9%)	13 (7.5%)
Use sunscreen	21 (9.1%)	52 (22.6%)	35 (15.2%)	72 (31.3%)	50 (21.7%)
Wear gloves	48 (20.9%)	82 (35.7%)	54 (23.5%)	35 (15.2%)	11 (4.8%)
Wear sunglasses	102 (44.3%)	57 (24.8%)	36 (15.7%)	24 (10.4%)	11 (4.8%)
Wear clothes that cover most of the body.	12 (5.2%)	69 (30%)	46 (20%)	39 (17%)	64 (27.8%)
Working in the early morning and afternoon hours	10 (4.3%)	47 (20.4%)	9 (30%)	68 (29.6%)	36 (15.7%)
Visiting your physician when observing suspicious symptoms	10 (4.3%)	35 (15.2%)	69 (30%)	76 (33%)	40 (17.4%)
Less sun exposure	7 (3%)	41 (7.8%)	71 (30.9)	72 (31.3%)	39 (17%)

**Fig. 1:**
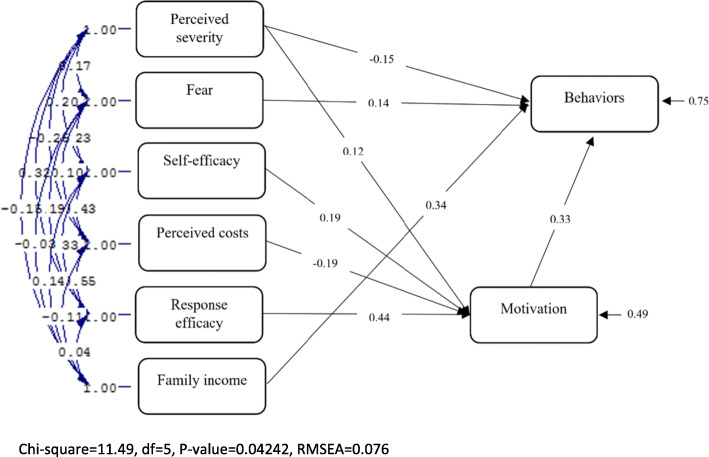
Path analysis model of PMT for skin cancer preventive behaviors. Rectangles: model constructs; Big arrows: path coefficient between the constructs; Small arrows: measurement arrows

The results of the path analysis indicated that PMT explains 51% of motivation variance and 25% of skin cancer preventing behaviors. Response efficacy construct was the most powerful predictor of the protecting motivation with 훽=0.44 and protecting motivation with a path coefficient of 0.33 was the most powerful predictor of skin cancer preventing behaviors. The self-efficacy constructs, perceived costs (inversely), and perceived severity significantly predicted motivation, and perceived severity and fear were predictors of these behaviors. The perceived vulnerability constructs and perceived rewards were not the predictors of motivation and behavior. However, household income, with a path coefficient of 0.34, was more powerful than all of PMT constructs in predicting the protection behaviors (Table 3, Fig. 1).

**Table 3:** Direct, indirect and total effects of PMT constructs on motivation and skin cancer preventing behaviors

Dependent variable	Independent variable	Direct effects	Indirect effects	Total effects
Motivation	Perceived severity	0.12*	–	0.12*
	Perceived costs	- 0.19*	–	- 0.19*
	Self-efficacy	0.19*	–	0.19*
	Response efficacy	0.44*	–	0.44*
Skin cancer preventing behaviors	Fear	0.14*	–	0.14*
	Perceived severity	- 0.15*	0.04	- 0.19*
	Perceived costs	–	- 0.06	- 0.06
	Self-efficacy	–	0.06	0.06
	Response efficacy	–	0.15*	0.15*
	Motivation	0.33*	–	0.33*
	Family income	0.34*	–	0.34*

*p < 0.05

Other variables including perceived severity, fear, perceived costs, response efficacy, and self-efficacy, excluding income variables, were also correlated. This indicates that the selection of variables was not mosaic form, but they rather interacted and the variables have been selected by attention to the theoretical model. The income variable had no significant correlation with fear and response efficacy. Two-way arrows and correlation coefficient amounts due to high numbers are not indicated in the figure. Table 4 indicates model fitting indices with acceptable indices values.

**Table 4:** Fitting indices resulted from path analysis of PMT in rural women

RMSEA	RMSR	IFI	NFI	AGFI	GFI	CFI	X2/df	df	X2
0.036	0.029	0.99	0.98	0.91	0.99	0.99	2.29	5	11.49

*RMSEA* Root Mean Square Error of Approximation

*RMSR* Root Mean Square Residual

*IFI* Incremental Fit Index

*NFI* Normed Fit Index

*AGFI* Adjusted Goodness of Fit Index

*GFI* Goodness of Fit Index

*CFI* Comparative Fit Index

Chi-square = 11.49, df = 5, *P*-value = 0.04242, RMSEA = 0.076

## Discussion

To the best of our knowledge, this is the first behavioral epidemiological study assessing the effective factors on skin cancer preventive behaviors among the Iranian rural women using PMT. Due to outdoor working, rural women are more exposed to sunlight and harmful UV radiation than other women. So they need to adopt more sun-protection behaviors than the usual population. The results of this study extend the knowledge obtained by previously performed studies on skin cancer preventive behaviors [22, 23]. The importance of this extension is in its effective role in developing the necessary information to design better interventional programs. This will finally lead to higher participation of rural women in screening and preventive programs. It is possible to present valuable services to rural women using simple educational, preventive, and screening measures. In other words, it is not necessary to deploy advanced diagnostics services and skin and cancer specialists in rural areas. The evidence indicated that investment in PHC is more efficient than advanced and expensive services [24].

The study results indicated that the rate of wearing sunglasses, gloves, and caps by rural women is lower than other preventing behaviors of skin cancer such as visiting a physician when observing suspicious symptoms, lower exposure to sunlight, using sunscreen, wearing sun-blocking clothes, and working in the early morning. The results of a study indicated that 18–29 years old Australian women wear sunscreen, gloves, caps, and sunglasses lower than other measures [25]. This result in rural women is in line with other Iranian study in rural men farmers in which a small proportion of them reported using sunscreen, hats, gloves, sunglasses, and protective clothing [26]. Low wearing protective equipment by rural women in the current study and also in the stated Australian women and Iranian rural men farmers may be an indicative of social and cultural obstacles that prevent using them.

The results of the path analysis indicated that PMT explains 51% of motivation variance and 25% of skin cancer preventing behaviors. Using this theory in Baghiani-moghadam et al. study on high school students has predicted 54% of motivation and 41% of skin cancer preventing behaviors [27]. Also, Dehbari et al. study on female university students predicted 39% of intention and 31% of sunlight protection behavior [28]. The difference in the prediction power of the theory may be due to the differences in studies population and statistics methods.

The results indicated that the motivation construct is the most powerful predictor of sunlight protecting behavior against skin cancer which is similar to other studies [25, 27]. This shows that motivation or intention to perform a behavior is a mediator between theory and behavior constructs. The role of protection motivation is undeniable in undertaking recommended skin prevention and control behaviors. Designing educational programs based on PMT can increase cancer protective behaviors [29].

The perceived severity and fear directly predict skin cancer preventing behavior reflecting that whatsoever people perceived severity of the disease, more they fear it which leads to adopting more preventive behaviors. The role of fear appeals in producing behavior changes is a proven fact [30]. However, this fear appeals don’t work in isolation and may cause defensive responding. So, it should be accompanied with efficacy messages [31].

Per capita income was the only demographic and background variable predicting skin cancer preventing behaviors. It seems that people with higher incomes are more likely to perform these behaviors. Low-income families, despite their good attitude and concerns about cancer, perform inadequate practices for cancer prevention [32]. Therefore, there is an urgent need for awareness and intervention raising programs throughout the country especially in the low-income regions to increase knowledge and behavior for skin cancer prevention and control. In this regard, insurance supports, providing services by the public sector and primary health care (PHC), and revising policies and programs are among the important measures to improve the access to the healthcare services by low socio-economic groups [33].

The most important construct which predicts the protection motivation or the intention of pursuing skin cancer prevention behaviors is response efficacy. Those who are aware of the efficiency and effectiveness of behaviors such as using sunscreen, cap, sunglasses, and wearing sun-blocking clothes have a more powerful intention to apply these behaviors. Studies by Zare-sakhvidi et al. and Rahaei et al. have indicated that this construct is one of the powerful predictors of protection motivation against cancers in adults [11, 13].

After response efficacy, self-efficacy was the most powerful predictor of motivation and intention to perform skin cancer preventive behaviors. In other words, those who are intended to perform these behaviors, in addition to those believing that these behaviors are effective in preventing skin cancer, are confident regarding their ability to perform these behaviors. Studies by Zare-sakhvidi et al. and Rahaei et al. have shown similar results [11, 13]. However, self-efficacy was a more powerful predictor than response efficacy in Zare-sakhvidi et al. study [11]. Self-efficacy, in addition to PMT, has been applied in other health behavior models including the health belief model [34, 35]. This indicates its effective role in the improvement of the predictive efficacy of healthcare models. Therefore, cancer and other diseases care providers should encourage self-confidence in patients and normal people to do the recommended health care and how to combat these diseases [36].

The results indicated that perceived costs significantly predict the protection motivation in a reverse manner. Each person’s estimation of protection behaviors costs can be a barrier to adopt protection behaviors. Zare-sakhvidi et al. obtained similar results, but this construct was not the predictor of protection motivation [11].

Collecting the questionnaire data through self-reporting is one of the study limitations. Thus the generalization of the results should be implemented by extra care. However, this problem can be resolved by giving enough time and fully explaining the study goals to the participants. A similar study by Bai et al. on the application of PMT in predicting intention to receive cervical cancer screening in rural Chinese women indicated that, if verified with longitudinal studies, PMT studies are applicable for intervention program development [14]. High participation of rural women in the study due to their interest to prevent skin cancer is among the study’s strengths.

## Conclusion

The results of this research indicated that PMT is a good framework to predict behavior especially in intention and motivation regarding skin cancer protection behaviors. The effective constructs on predicting skin cancer preventive behaviors, in addition to motivation, were response efficacy, self-efficacy, and perceived severity (directly), and perceived costs (reversely). Also, the household income was a relatively strong predictor to adopt sunlight protection behaviors to avoid skin cancer. It is thus recommended to employ this theory and its constructs to design interventional programs to promote skin cancer preventive behaviors.

## Data Availability

The datasets analyzed during the current study are available from the corresponding author on reasonable request.

## Abbreviations

PMT: protection motivation theory
UV: ultra violet
CVR: content validity ratio
CVI: content validity index
CFI: comparative fit index
GFI: goodness of fit index
AGFI: adjusted goodness of fit index
NFI: normed fit index
RMSEA: root mean square error of approximation
RMSR: root mean square of residuals
LISREL: linear structural relations model
PHC: primary health care
IFI: incremental fit index
USD: United States Dollar
